# The Characterisation of *Lactococcus garvieae* Isolated in an Outbreak of Septicaemic Disease in Farmed Sea Bass (*Dicentrarchus labrax*, Linnaues 1758) in Italy

**DOI:** 10.3390/pathogens13010049

**Published:** 2024-01-04

**Authors:** Cristian Salogni, Cristina Bertasio, Adelchi Accini, Lucia Rita Gibelli, Claudio Pigoli, Francesca Susini, Eleonora Podavini, Federico Scali, Giorgio Varisco, Giovanni Loris Alborali

**Affiliations:** 1 Istituto Zooprofilattico Sperimentale della Lombardia e dell’Emilia Romagna “Bruno Ubertini”, 25124 Brescia, Italy; cristian.salogni@izsler.it (C.S.); cristina.bertasio@izsler.it (C.B.); luciarita.gibelli@izsler.it (L.R.G.); claudio.pigoli@izsler.it (C.P.); eleonora.podavini@gmail.com (E.P.); giorgio.varisco@izsler.it (G.V.); giovanni.alborali@izsler.it (G.L.A.); 2 Skretting Italia S.p.A., 37060 Mozzecane VR, Italy; adelchi.accini@skretting.com; 3 Istituto Zooprofilattico Sperimentale del Lazio e della Toscana “M. Aleandri”, 00178 Roma, Italy; francesca.susini@izslt.it

**Keywords:** Mediterranean, aquaculture, environment, lactococcosis, sea bass, septicaemia

## Abstract

In aquaculture, *Lactococcus garvieae* is a common fish pathogen that can cause significant economic losses in several fresh and saltwater species. Despite the extensive range of hosts, *L. garvieae* infection in sea bass (*Dicentrarchus labrax*) has rarely been reported. During the summer of 2023, an outbreak occurred in an inland farm in the Gulf of Follonica (Tuscany, Italy). Fish of various sizes were affected, showing apathy, inappetence, erratic swimming and eye lesions, while the mortality was low (2–3% per month). Anatomopathological examinations suggested a septicaemic infection characterised by melanosis, diffuse redness (skin and fins), paleness (gills and internal organs), haemorrhages and splenomegaly. Seventy swabs from the viscera of 14 subjects were collected and colonies similar to *Streptococcus* spp. grew from all the samples. *Lactococcus garvieae* was identified via the biochemical tests, API20STREP, MALDI-TOF, 16S rDNA and whole genome sequencing. Genetical characterisation revealed remarkable differences between this isolate and the strains previously isolated in Italian fish farms. Feed treatments with flumequine and erythromycin were ineffective. Considering the limited effects of antimicrobials, preventive measures, such as vaccination and biosecurity, should be implemented.

## 1. Introduction

*Lactococcus garvieae* is a Gram-positive bacterium that causes lactococcosis, a septicaemic–haemorrhagic infection in fish. It is a major bacterial pathogen in the aquaculture sector that is widespread worldwide and responsible for significant economic losses in fish farming [1].

Lactococcosis in fish typically begins with the affected individuals becoming anorexic and showing abnormal behaviour such as erratic and spiralling swimming. The progression of the disease leads to a swollen abdomen, anal prolapse, exophthalmia and cataracts. Internal organs become congested, the spleen and liver are enlarged, ascitic fluid accumulates in the peritoneal cavity and exudates may be found in the brain. In the most severe cases, the affected fish show extensive haemorrhages and necrosis of the organs with signs of ulceration and eye loss [2].

*Lactococcus garvieae* can infect a wide range of farmed and wild fish species, in both fresh and salt water, especially when the water temperature is above 15 °C [3], the most representative being Japanese eel (*Anguilla japonica*), Nile tilapia (*Oreochromis niloticus*), clown coris (*Coris aygula*), spotted sorubim (*Pseudoplatystoma corruscans*), olive flounder (*Paralichthys olivaceus*), greater amberjack (*Seriola dumerili*), yellowtail (*Seriola quinqueradiata*), rainbow trout (*Oncorhynchus mykiss*), flathead grey mullet (*Mugil cephalus*) and wels catfish (*Silurus glanis*) [4]. The list of affected species is still growing and includes not only fish but also invertebrates such as freshwater shrimp (*Macrobrachium rosenbergii*) and octopus (*Octopus vulgaris*). Mammals such as dolphin (*Tursiops truncates*) [5], cattle, buffalo, pig and human can also be affected [6]. This increasing range of hosts has led researchers to consider *L. garvieae* as an emerging opportunistic pathogen [6]. *Lactococcus garvieae* has also shown the ability to adapt and evolve over time under different conditions [7]. For example, *L. garvieae* isolated from farmed greater amberjack in Japan have, since 2012, revealed the presence of different serotypes and genotypes [7]. Furthermore, the specific genetic identification of streptococcal infections has become necessary since the recent isolation of *Lactococcus petauri*, *Lactococcus lactis* subsp. *lactis*, *L. lactis* subsp. *cremoris* and *L. lactis* subsp. *tructae* [8,9,10].

Despite its adaptability and wide range of potential hosts, and despite the fact that it has been demonstrated under experimental conditions, *L. garvieae* infection in sea bass (*Dicentrarchus labrax*) [11] has been reported only once in the field [12]. Sea bass is a widespread fish species with high commercial importance in the Mediterranean Basin [13]. With a production of 7400 tonnes in 2022, Italy is the fifth largest producer in the world after Turkey, Greece, Spain and Egypt [14]. Sea bass was the first non-salmonid species to be farmed on a commercial scale in Europe, as early as the 1960s; Italy, together with France, were the first countries to develop effective seabass production techniques [15]. The majority of production comes from aquaculture, with fisheries playing a secondary role [16]. Since the 1990s, Italian fish farming has shifted from a traditional land-based aquaculture system (valliculture) to land-based ones and sea cage offshore farms (floating cages in the sea) [17,18]. This change has led to an increase in production, but has also raised some concerns about water pollution, changing coastal habitats, disturbing wildlife and reducing the attractiveness of tourist sites [19,20,21,22,23].

In this case report, we describe an outbreak of *L. garvieae* infection in sea bass reared in the Gulf of Follonica (Tuscany, Italy), a rare event occurring in an area characterised by a high density of fish farms.

## 2. Case Presentation

In an inland intensive sea bass farm located in the Gulf of Follonica (Tuscany, Italy), juvenile and adult fish showed signs of disease characterised by apathy, inappetence, erratic swimming just below the water surface and visible pop-eyes or the enucleation of the ocular globe. The skin of the affected fish appeared reddish in the ventral and jugular areas. All subjects gave their informed consent for the use of the animal samples submitted to IZSLER for research and publication purposes.

The water for the farm was supplied by pumping seawater directly from the seacoast, and the sea bass were raised in concrete flow-through raceways, receiving surface water reaching temperatures of 24–25 °C during summer.

Recurrent summer episodes of increased mortality and similar clinical signs of vibriosis or photobacteriosis have been reported over the last five years in the same farm. Mortality started in late August, when the temperature of the inlet water was raised over 23 °C, reaching a maximum temperature of 25 °C, affecting approximately 8% of the farmed fish. Mortality was low, with an increase in the average monthly mortality rate of 2 to 3% (September). This increase was stable throughout the outbreak, with no significant peak. Sea bream (*Sparus aurata*) were also present at the farm and showed no signs of disease or increased mortality during the outbreak.

Seventy swabs from the internal viscera (brain, kidney, liver, eye and spleen) of 14 recently deceased sea bass were aseptically collected for bacteriological examination. The fish were transported to the laboratory, refrigerated at melting ice temperatures, and samples were collected less than 24 h after the subjects were found dead. The fish showed rigor mortis at the time of sampling and no other cadaveric alterations. The sampled subjects were juveniles and adults with a mean length of 16.5 cm (standard deviation: 6.8 cm; range: 12–35 cm) and a mean weight of 72 g (standard deviation: 112 g; range: 21–391 g). In addition to the bacteriological analyses, routine anatomopathological and parasitological (wet mounts of skin, gills and intestine) examinations were performed for all 14 subjects. Viral encephalopathy and retinopathy were also researched with an end-point rt-PCR test [24]. Parasitological and virological analyses were negative.

At the time of the sampling (September 2023), the stocking density was 33 kg/m^3^. The water temperature was 24 °C, the pH was 8.29 and the salinity was 37.5‰. Unionised ammonia and nitrate levels were below the thresholds considered harmful to sea bass.

### 2.1. Anatomopathological Examinations

**Figure 1:**
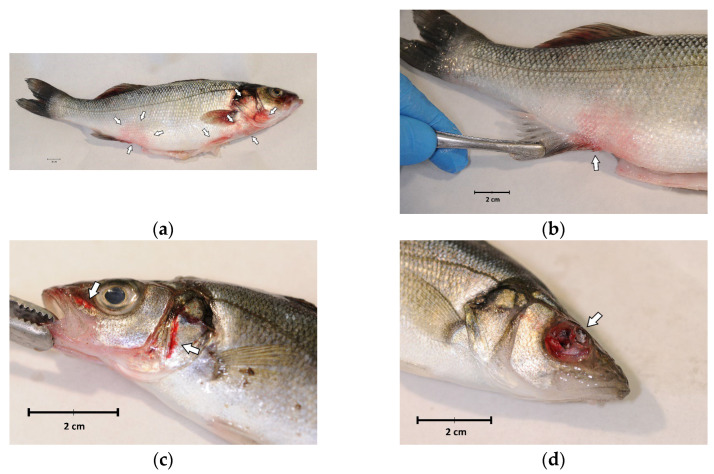
External examination of sea bass (*Dicentrarchus labrax*) involved in the outbreak of lactococcosis caused by *Lactococcus garvieae*: (**a**) External aspect of a sea bass with *L. garvieae* septicaemia characterised by diffuse skin and fin redness (arrows); (**b**) reddish area on the side, near the pelvic fin; (**c**) haemorrhages of the head (arrows); (**d**) ulceration and eye loss.

External examination revealed a good body weight, but all fish showed melanosis, diffuse redness of the skin and fins (Figure 1a,b) and haemorrhages (Figure 1c). Ulceration or eye globe loss (Figure 1d) were evident in 4 out of 14 individuals (28.6%) (Figure 1).

**Figure 2:**
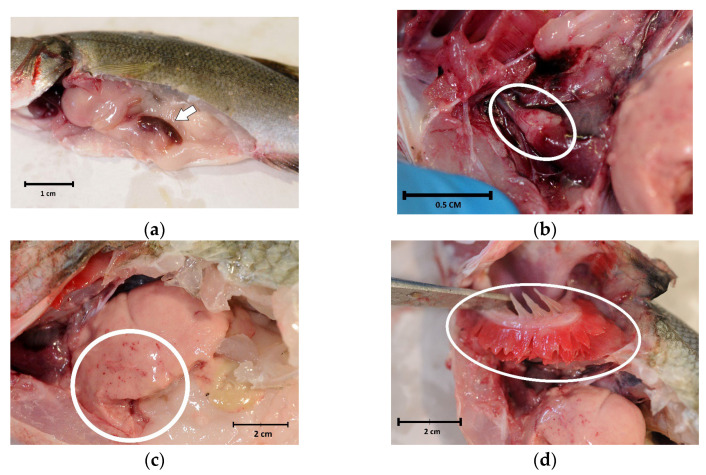
Internal examination of sea bass (*Dicentrarchus labrax*) involved in the outbreak of lactococcosis caused by *Lactococcus garvieae*: (**a**) paleness and splenomegaly (arrow); (**b**) pericarditis and cardiac haemorrhages (oval); (**c**) liver haemorrhages (circle); (**d**) paleness of the gills (oval).

Internal examinations revealed marked splenomegaly (Figure 2a) in 10 fish (71.4%), cardiac haemorrhages (Figure 2b) in 4 (28.6%) and liver haemorrhages (Figure 2c) in 2 (14.3%). Evident paleness of the viscera (Figure 2a) and gills (Figure 2d), strongly suggestive of anaemia, were only present in fish with eye loss.

**Figure 3:**
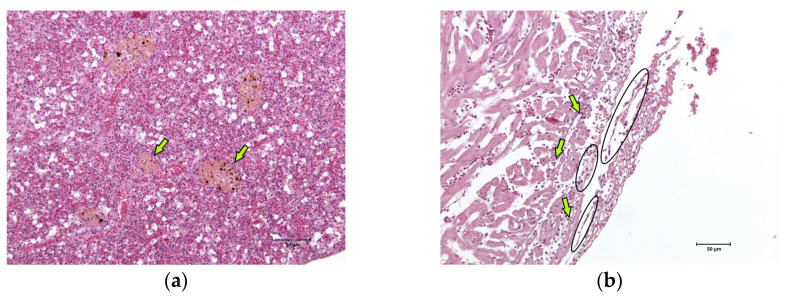
Histological images (haematoxylin–eosin stain, 20× magnification) of the sea basses (*Dicentrarchus labrax*) involved in the outbreak of lactococcosis caused by *Lactococcus garvieae*: (**a**) spleen. In the spleen, hyperplasia of the melanomacrophage centres (examples marked with arrows) associated with severe and diffuse white pulp depletion was observed; (**b**) heart. In the heart, multifocal fibrinous (examples marked with ovals) pericarditis with haemorrhages (examples marked with arrows) was observed.

The skin, gills, brain, liver, kidney, spleen, and heart of two fish were sampled and fixed in 10% neutral buffered formalin for a routine histological analysis. After paraffin embedding, 5 µm sections were obtained from each sample and haematoxylin–eosin stained. Major lesions were found in spleen and cardiac tissues. In the spleen, hyperplasia of the melanomacrophage centres associated with the severe and diffuse depletion of the white pulp was observed (Figure 3a). Multifocal fibrinous pericarditis with haemorrhages was also noted (Figure 3b).

### 2.2. Bacteriological and Biochemical Tests

Seventy swabs were inoculated in Columbia blood agar (CA, 5% mutton red blood cells), tryptic soy agar (TSA) and thiosulfate citrate bile salt sucrose agar (TCBS) and incubated under aerobic conditions at 25 °C for 48 h. After two days, the plates showed abundant growth on both the CA and TSA from all samples. Colonies were whitish, umbonate microhaemolytic (up to 1 mm diameter) and α-haemolytic, similar to that of the genus *Streptococcus*. No other kind of colonies grew, so the infection was considered pure and septicaemic. Colonies were re-inoculated onto TSA and CA media at 25 °C for 48 h. Purified strains obtained from fish in group 1 (LI296620A_23) and group 2 (LI296620B_23) were submitted for phenotypic and genetic investigation.

Strains were typed according to their phenotypical (haemolytic activity, Gram stain, Indian ink stain) and biochemical (macro method tests and miniaturised Analytical Profile Index (API) 20 STREP system) characteristics. A matrix-assisted laser desorption ionisation (MALDI) time of flight (TOF) mass spectrometry analysis (Vitek MS Plus, Biomerieux, France) was also performed.

Gram staining showed the presence of Gram-positive cocci organised in short chains or pairs. The biochemical tests identified the species of the bacterium as *L. garvieae*. Specifically, biochemical tests were performed to determine differences with *Streptococcus* spp. as follows: growth on the CA medium at different temperatures (20 °C, 37 °C and 42 °C), in a saline environment (TSA, 6.5% NaCl), on MacConkey agar and on a motility medium. Oxidase and catalase tests were also performed. In Table 1, the results of the biochemical tests are shown and compared with other *L. garvieae* strains identified by our lab over the years from different outbreaks with high mortality in rainbow trout and from a sea bass reared in an offshore farm in the Gulf of Follonica (strain LI329384 _23). The latter did not come from any outbreak but was isolated at the end of July 2023 from samples that were collected during the farm’s routine monitoring.

Despite certain discrepancies, the biochemical profile acquired through the API 20 strep corresponded to earlier findings from *L garvieae* strains from rainbow trout in Italy (Table 1). However, the code recognised multiple taxa since there is not yet a biochemical profile exclusively specific to *L. garvieae* in any of the automated devices [25]. Strain LI296620A_23 was analysed with MALDI TOF and identified as 99.90% similar as *L. garvieae*. Main spectrum profiles are reported in Figure S1.

### 2.3. Molecular Analysis

Partial sequencing of 16S ribosomal DNA (rDNA) was executed on strain LI296620A_23 using a Fast MicroSEQ^TM^ 500 16S rDNA PCR kit (Thermo Fisher Scientific, Waltham, MA, USA) according to Patel et al. (2000) [26]. Details of the method are reported in Table S1.

The forward and reverse sequences were assembled using SeqMan ultra 17 (DNASTAR) and the consensus sequence was queried against the public databases through BLAST [27] (accessed on 15 November 2023). The best hit was with the *L. garvieae* strain JJJN1 (CP026502.1), a strain isolated from redlip mullet (*Liza haematocheila*) in South Korea in 2017.

To better characterise the strains, all the isolates from sea bass from the Gulf of Follonica (LI296620A_23, LI296620B_23, LI329384_23) were subjected to whole genome sequencing (WGS). In addition, considering the phenotypical similarity among *L. garvieae* previously isolated from farmed rainbow trout in Northern Italy, a group of eight *L. garvieae* strains (BS000031_98, NO346039_22, NO358429_21, PV358422_21, VR143326_21, NO317338_20, TV252859_20, SO223838_20) were selected and submitted to WGS in parallel with the sea bass samples. Briefly, the genomic DNA was extracted from isolated colonies using the NucleoSpin Tissue kit (Macherey Nagel, Duren, Germany). The concentration of genomic DNA was quantified using a QuantiFluor^®^ ONE dsDNA kit and a Quantus Fluorometer (Promega, Madison, WI, USA). Illumina sequencing libraries were generated with an Illumina DNA Prep (M) tagmentation kit and sequenced on an Illumina MiniSeq platform with 2 × 150-bp paired-end reads (Illumina, San Diego, CA, USA), following the manufacturer’s protocol. The reads were trimmed with Trimmomatic v0.39 [28] and assembled de novo using SPAdes v3.15.4 [29] with default parameters. The quality of the reads was assessed using FastQC v0.11.9 (https://www.bioinformatics.babraham.ac.uk/projects/fastqc/ accessed on 23 October 2023) and that of the assemblies with Quast v5.2 [30]. The absence of contamination was assessed using Kraken2 available on the BV-BRC platform [31]. The raw reads were available on the SRA database under the project PRJNA1041340.

**Figure 4:**
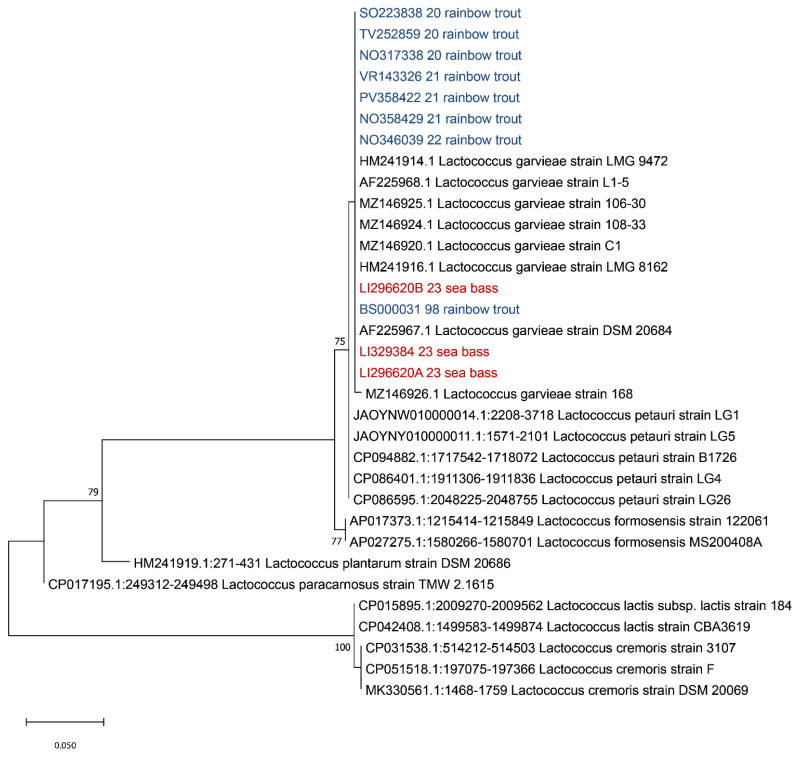
Phylogenetic analysis based on the ITS 16S-23S region. Strains isolated from sea bass (*Dicentrarchus labrax*) described in this manuscript are highlighted in red while the isolates from previous outbreaks that occurred in Italy in rainbow trout (*Oncorhynchus mykiss*) are highlighted in blue. The evolutionary history was inferred using the Maximum Likelihood method and Tamura–Nei model. Bootstrap values are reported near the nodes. The tree with the highest log likelihood (−699.15) is shown. The percentage of trees in which the associated taxa clustered together is shown next to the branches. The tree is drawn to scale, with branch lengths measured in the number of substitutions per site. This analysis involved 33 nucleotide sequences. There were a total of 262 positions in the final dataset.

Since many authors reported that an analysis of the 16s rDNA gene is not able to discriminate between *L. petauri* and *L. garvieae* [32,33,34,35], the 16S-23S ITS region sequence was extracted from the obtained contigs and aligned with sequences of *Lactococcus* spp. retrieved from Genbank (accession numbers of *L. garvieae*: HM241914, AF225968, AF225967, MZ146926, MZ146925, MZ146924, MZ146920 and HM241916; *L. petauri*: JAOYNW010000014, JAOYNY010000011, CP094882, CP086401 and CP086595; *L. formosensis*: AP017373 and AP027275; *L. plantarum:* HM241919; *L. paracarnosus*: CP017195; *L. lactis*: CP015895 and CP042408; and *L. cremoris*: CP031538, CP051518 and MK330561). Evolutionary analyses were conducted in MEGA X using the Maximum Likelihood method and Tamura–Nei model [36,37], with a bootstrap of 1000, obtaining the tree reported in Figure 4.

As shown in Figure 4, based on the ITS 16S-23S region, all our strains clustered with *L. garvieae*, in agreement with previous results of the 16S rDNA region. In order to identify the serotype of the isolated *L. garvieae*, an in silico analysis was performed using the two primer sequences described by Ohbayashi and colleagues in 2016 [38], obtaining a fragment of 285 bp, which is compatible with serotype I.

**Figure 5:**
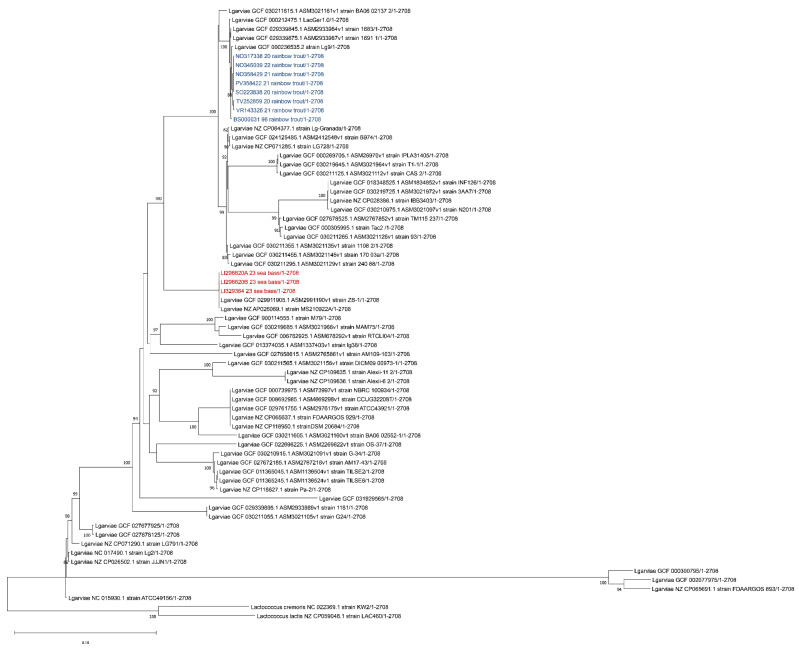
SNP tree resulting from a CSI-Phylogeny tool. Strains isolated from sea bass (*Dicentrarchus labrax*) described in this manuscript are highlighted in red while the isolates from previous outbreaks that occurred in Italy in rainbow trout (*Oncorhynchus mykiss*) are highlighted in blue. *Lactococcus lactis* strain LAC460/1 and *Lactococcus cremoris* strain KW2/1 were used as the outgroups.

The assemblies of the three strains isolated from the sea bass and of the eight from the rainbow trout were compared whilst also considering the sequences retrieved from Genbank using a CSI-Phylogeny 1.4 tool [39] (available on https://cge.food.dtu.dk/services/CSIPhylogeny/ accessed on 21 December 2023). Briefly, this tool calls and filters the SNPs, performs site validation and infers a phylogeny based on the concatenated alignment of the high-quality SNPs. The genome of *L. garvieae* strain_ATCC 4915 (accession number: NC_015930.1) was used as a reference. The resulting phylogenetic tree was processed using MegaX (Figure 5).

As shown in Figure 5, the strains isolated from sea bass were different to those from Italian farmed rainbow trout and clustered with the *L. garvieae* strain MS210922A (accession number: NZ_AP026069.1) isolated in 2021 from farmed greater amberjack in Japan [7] and from the *L garvieae* strain ZB-1 (accession number: GCF_029911905.1) isolated in 2022 in Fuzhou, China, from a large yellow croaker (*Larimichthys crocea*) with 0 SNPs (Single Nucleotide Polymorphisms) of difference (Table S2). Further in silico analyses were performed to screen the presence of virulence-related genes in the sea bass strains. The presence of the 16.5 kb capsule gene cluster described by Morita et al. [40] was investigated using Geneious Prime software, version 2023.2; in all these strains, this genomic region was absent. The strains were also investigated for the presence of other genes related to virulence, such as those codifying haemolysin *(hlyIII)*, the fibronectin-binding protein *(fpb)*, penicillin acylase *(pva)* and bile salt hydrolase *(bsh1* and *bsh2),* using the sequences reported by Eraclio et al. [41] or their comparisons. The results are shown in Table 2.

In order to detect any mutations, the nucleotide sequences of those genes of the sea bass strains were aligned with the corresponding genes of the Lg2 strain using BioEdit software, version 7.2 [42]. All the strains from sea bass revealed the same nucleotide sequence, while, with respect to the virulent strain Lg2, they showed some SNPs in all three of the genes (*hlyIII* 63 T > C, 66 C > T, 117 G > A, 133 G > C, 567 C > T, 585 T > C, 600 T > G; *fpb* 87 G > A, 186 G > T, 297 A > G, 372 G > A, 459 C > T, 480 G > T, 591 C > T, 852 A > T, 993 A > C, 1392 C > T, 1515A > G; *pva* 207 A > G, 349 T > C, 359 G > A, 468 T > C, 489 T > C, 561C > T, 575 C > T). In terms of aminoacidic composition, all the SNPs were identified as synonymous, except for *hlyIII* 133 G > C, which determined the substitution of the amino acid (aa) 45 (Ala > Pro) in the haemolysin protein, and for *pva* 349 T > C and 575 C > T, which caused the substitutions of aa 117 (Phe > Leu) and aa 192 (Ser > Leu), respectively.

### 2.4. Antimicrobial Therapy and Susceptibility Tests

Assuming an infection caused by *Vibrio* spp. or *Photobacterium* spp. on the basis of clinical signs, a first cycle of treatment with flumequine at 12 mg/kg of body weight (BW) for five days was carried out using medicated feed without significant results.

After the identification of *L. garvieae* as a causative agent, a disc diffusion method on Mueller–Hinton agar (Oxoid, Basingstoke, UK) [43] was employed to conduct an antimicrobial susceptibility test. This utilised the Mueller–Hinton solid medium supplemented with mutton red blood cells (5%). A bacterial suspension, with a turbidity of 0.5 McFarland, was inoculated and anaerobically incubated for 48 h at 25 °C, according to the standard protocols of the Clinical and Laboratory Standard Institute (CLSI) [44]. Since the breakpoints specific to fish were not available, we reported those of mammals following the CLSI guidelines [45]. The microorganism was susceptible to amoxicillin and clavulanic acid (30 µg), ceftiofur (30 µg), cephalothin (30 µg), erythromycin (15 µg), florfenicol (30 µg), tetracycline (30 µg) and thiamphenicol (30 µg). Resistance was found against ampicillin (10 µg), enrofloxacin (5 µg), kanamycin (30 µg), oxacillin (1 µg), penicillin (10 µg), pirlimycin (2 µg) and trimethoprim and sulphonamide (25 µg).

On the basis of the antimicrobial susceptibility test, a second treatment, again with medicated feed, was administered with erythromycin at 75 mg/kg of BW for ten days. Mortality began to decrease from the third day of therapy but rapidly rose again, soon after the end of treatment.

## 3. Discussion

*Lactococcus garvieae* was reported for the first time as a fish pathogen in yellowtail in Japan in 1974 and was initially known as *Enterococcus seriolicida*. In Europe, the first account of *L. garvieae* infection in fish dates back to 1993 [46,47]. Since then, this bacterium has been isolated in several fish species, becoming one of the most important pathogens in the last decade. *Lactococcus garvieae* infections are difficult to control with antimicrobials and have caused massive losses in both marine and freshwater farmed fish worldwide [1,48,49,50]. In Italy, *L. garvieae* has been mainly found in rainbow trout, especially during the warm season when the temperature rises above 15 °C. Infections in rainbow trout farms can be severe, with septicaemia and high losses with over 20% mortality each outbreak if appropriate therapy is applied. Outbreaks may recur during the same season and during subsequent years, causing economic losses that can exceed 50–80% of the total production in several trout farms, considering the annual cumulative mortality [51,52,53,54]. Despite its rapid spread among trout farms and its poor species specificity, the infection has been limited to rainbow trout or other salmonids like brook trout (*Salvelinus fontinalis*) [54].

*Lactococcus garvieae* can enter and spread throughout fish farms in different ways: with incoming water, by persistence of the pathogen in the sediment, by faecal transmission, by the introduction of carrier fish through influent water or by dead or dying infected individuals [3]. Where biosecurity barriers are low or limited, such as in Mediterranean seawater aquaculture where fish are mainly kept in open water, the risk of introducing pathogens with water is high and second only to the introduction of new fish from uncontrolled external sources [55,56]. For this outbreak, the most likely source of the infection was inlet water that came from a coastal intake without any kind of treatment. The farm was supplied with surface water, which is a known risk factor, especially when infected wild fish are present in the area [48]. Alternative sources of infection, such as the introduction of infected fish or contamination due to changes in feed management, were unlikely. The disease had occurred in fish of different sizes and there was no evidence of fingerlings or juveniles being imported from infected farms or areas with known outbreaks.

Genome sequencing provided interesting data on the potential source of infection. The strains isolated during the outbreak were different from those circulating in Italian trout farms (194–196 SNPs and Table S2), but identical (0 SNPs) to strains recently found in China and Japan. These results excluded transmission from the classical host in Italy (trout) to sea bass. On the other hand, the 100% identity match between the Chinese and Japanese strains was surprising since, to the best of our knowledge, there seemed to be no epidemiological link with the Gulf of Follonica.

The clinical characteristics of this *L. garvieae* outbreak were not distinguishable from those of other sea bass septicaemic diseases like vibriosis, photobacteriosis or aeromoniasis. The outbreak was also similar from an epidemiological perspective, given the role of water temperature in the onset of the disease. In other species, the severity and mortality of lactococcosis can greatly vary depending on the species affected, the age of the fish, the virulence of the strain, the route of infection, as well as environmental and water quality factors [1]. This outbreak in sea bass was different to those described in Italy before, which have always involved trout. The severity and mortality were lower, but the disease affected various age groups; in the case of trout, however, it was restricted to adults in the finishing stage [51,52,53,54].

Although the pathogenic mechanism of *L. garvieae* is poorly understood, the presence of a capsule represents a known virulence factor in fish infection [40,57,58]. Nevertheless, in Asia, where vaccination is widely used in the farming of seawater fish, recently isolated strains showed the modification or even complete loss of such a capsule [7]. In addition to the capsule, a recent comparative genome analysis on *L. garvieae* strains from different sources (diseased fish, foods, environment, etc.) identified other potential virulence factors, such as adhesion surface proteins, haemolysins and bile salt hydrolase [41]. In this outbreak, the isolated strain lacked the capsule gene cluster but possessed the genes encoding for three putative virulence factors (haemolysing, fibronectin-binding protein and penicillin acylase).

In addition to virulence factors, environmental stressors can also contribute to an increased severity and mortality of *L. garvieae* infections. In this case, intensive farming conditions and high water temperatures were likely to have played a major role in the onset and maintenance of the outbreak. The latter is particularly known to be a major risk factor. Indeed, several studies reported a relationship between water temperature and *L. garvieae* infections [2]. This is of particular concern considering that the water temperature in the Gulf of Follonica exceeds 24–25 °C during the summer, with the water also stagnating as a result of the persistence of calm and sunny days. The steady increase in water temperatures over the years poses a serious threat to Mediterranean aquaculture, favouring the negative effects on the movement of fish stocks, the diffusion of transboundary aquatic infections and increasing the negative anthropogenic effects on the marine habitat [59,60,61,62,63,64]. The spread of infectious pathogens can also cause an increase in the use of antimicrobials, which leads to the selection of resistant strains [65,66].

Mortality was relatively low, but it is difficult to determine the cause. There is currently no information available on the mortality under the field conditions for comparison. In the first study in which sea bass were experimentally infected with *L. garvieae*, no mortality or clinical signs were observed [67]. However, only juveniles were challenged and with a strain different from the one causing this outbreak. In contrast, a recent study that involved older fish, also under experimental conditions, reported high mortality and severe clinical signs [11]. Mortality in this outbreak may have been influenced by a natural resistance of the sea bass to the pathogen, the absence of some virulence factors (e.g., the capsule) in the strain involved and also other factors such as high temperature and stocking density. Histopathological examinations also revealed features partially comparable with those described in previous experimental infections. The agent was isolated from the liver and kidney where histological examination showed no relevant lesions, as reported by Türe and colleagues [67], while the lesions found in the spleen and heart tissue were similar to those described by Akaly et al. [11]. These authors also described severe anaemia in the late stages of the infection associated with hyperaemia and haemorrhaging in the internal organs [11]. In our case, severe paleness of the viscera and gills, suggesting anaemia, was only observed in association with eye loss. This difference may have been attributed to variations in field conditions and a shorter duration of the infection.

The control of the disease is based on antimicrobial therapy and vaccination with an intraperitoneal bacterin vaccine [68]. However, data on *L. garvieae* infection in saltwater fish farming in the Mediterranean area are scarce and an outbreak in sea bass has never been described, so no information is available on the effectiveness of such control strategies in this context. In this case, the mortality decreased during the second antimicrobial therapy but rapidly rose again soon after the end of treatment. Persistent environmental conditions favourable to infection and the low feed intake of the anorexic fish may have induced relapse. Furthermore, the ten-day course of therapy with a macrolide (erythromycin) could have been prolonged with beneficial effects. However, caution should be exercised when extending such treatments as macrolides are considered by the WHO highest priority critically important antimicrobials for human medicine [69].

Considering the limited effects of the antimicrobial treatments, the risks related to antimicrobial resistance and the tendency of the microorganism to engender recurrent outbreaks during the hot season, preventive measures, such as vaccination based on recently isolated and characterised strains, should be implemented to improve fish welfare, and biosecurity should be increased where possible. At the time of this report, the potential carriers of the infection outside the farm were not known, and future investigations should be carried out on the wild fish population, particularly given the similarity with Chinese and Japanese strains. Finally, considering the scarcity of information available, further investigation should be conducted on the relationship between the agent and host, for example, by also sampling healthy subjects during an outbreak and carrying out challenge tests under controlled conditions using strains isolated in the field.

## Figures and Tables

**Table 1:** Phenotypic characterisation of *Lactococcus garvieae* strains isolated from sea bass (*Dicentrarchus labrax*) during an outbreak in an inland intensive farm located in the Gulf of Follonica (Tuscany, Italy) compared with a strain isolated from sea bass in an offshore farm in the same area and strains previously isolated in Italy from rainbow trout (*Oncorhynchus mykiss*).

Parameter	LI296620A_23	LI296620B_23	LI329384_23	BS000031_98 NO317338_20 TV252859_20 NO358429_21 PV358422_21 VR143326_21 NO346039_22	SO223838_20
Country of isolation	Italy	Italy	Italy	Italy	Italy
Year(s) of isolation	2023	2023	2023	1998, 2020–2022	2020
Farm Type	Inland saltwater	Inland saltwater	Offshore saltwater	Inland freshwater	Inland freshwater
Affected species	Sea bass	Sea bass	Sea bass	Rainbow trout	Rainbow trout
Mortality ^1^	+	+	−	+++	++
Gram	+	+	+	+	+
Morphology	Ovoid	Ovoid	Ovoid	Ovoid	Ovoid
Motility	−	−	−	−	−
Catalase	−	−	−	−	−
Oxidase	−	−	−	−	−
Oxidation (O)—Fermentation (F)	F	F	F	F	F
Citrate	−	−	−	−	−
DNase	−	−	−	−	−
Vogues-Proskauer	+	+	+	+	+
Esculin	+	+	+	+	+
Hippurate	−	−	−	+	+
Pyrrolidonyl arylamidase	+	+	+	+	+
α-galactosidase	−	−	−	−	−
β-glucuronidase	−	−	−	−	−
β-galactosidase	−	−	−	−	−
Phenylalanine ammonia lyase	−	−	−	−	−
Leucyl aminopeptidase	+	+	+	+	+
Arginine dihydrolase	+	+	-	+	+
Ribose	+	+	+	+	+
Arabinose	−	−	−	−	−
Mannitol	+	+	−	+	+
Sorbitol	−	−	−	−	−
Lactose	−	−	−	−	−
Trehalose	+	+	+	+	+
Inulin	−	−	−	−	−
Raffinose	−	−	−	−	−
Starch	−	−	+	−	−
Glycogen	−	−	−	−	−
β-haemolysis	−	−	−	−	−
API CODE	5,143,110	5,143,110	5,143,011	7,143,110	7,143,110

^1^ Mortality: (+++) = high, >20%; (++) = medium; 10–20%; (+) = low, >10%; (−) = no increase in baseline mortality.

**Table 2:** In silico detection of putative virulence genes in sea bass *Lactococcus garvieae* strains.

Strain	hlyIII ^1^	Fbp ^1^	Pva ^1^	bsh1 ^1^	bsh2 ^1^
LI296620A_23	+	+	+	−	−
LI296620B_23	+	+	+	−	−
LI329384_23	+	+	+	−	−

^1^ (+) = gene detected, (−) = gene not detected.

## Data Availability

Data are contained within the article or supplementary material. The strain isolated during the study is available from the corresponding author on reasonable request.

## Supplementary Materials

The following supporting information can be downloaded at: https://www.mdpi.com/article/10.3390/pathogens13010049/s1, Table S1: Partial sequencing of 16S ribosomal DNA (rDNA) on the strain LI296620A_23 (method details). Table S2: SNP Matrix. Figure S1: Main spectrum profiles of the strain LI296620A_23.
