# Supplementary material for: The Characterisation of Lactococcus garvieae Isolated in an Outbreak of Septicaemic Disease in Farmed Sea Bass (Dicentrarchus labrax, Linnaues 1758) in Italy
Source: Pathogens. 2024 Jan 4;13(1):49. doi: 10.3390/pathogens13010049 (PMC10820654; doi:10.3390/pathogens13010049)
Supplement: Supplementary file 1 [file pathogens-13-00049-s001.zip › Supplementary_Figure_S1.pdf]

Figure S1 – Salogni et. al – The Characterization of *Lactococcus garvieae* isolated in an Outbreak of Septicaemic Disease in Farmed Sea Bass (*Dicentrarchus labrax*, Linnaues 1758) in Italy

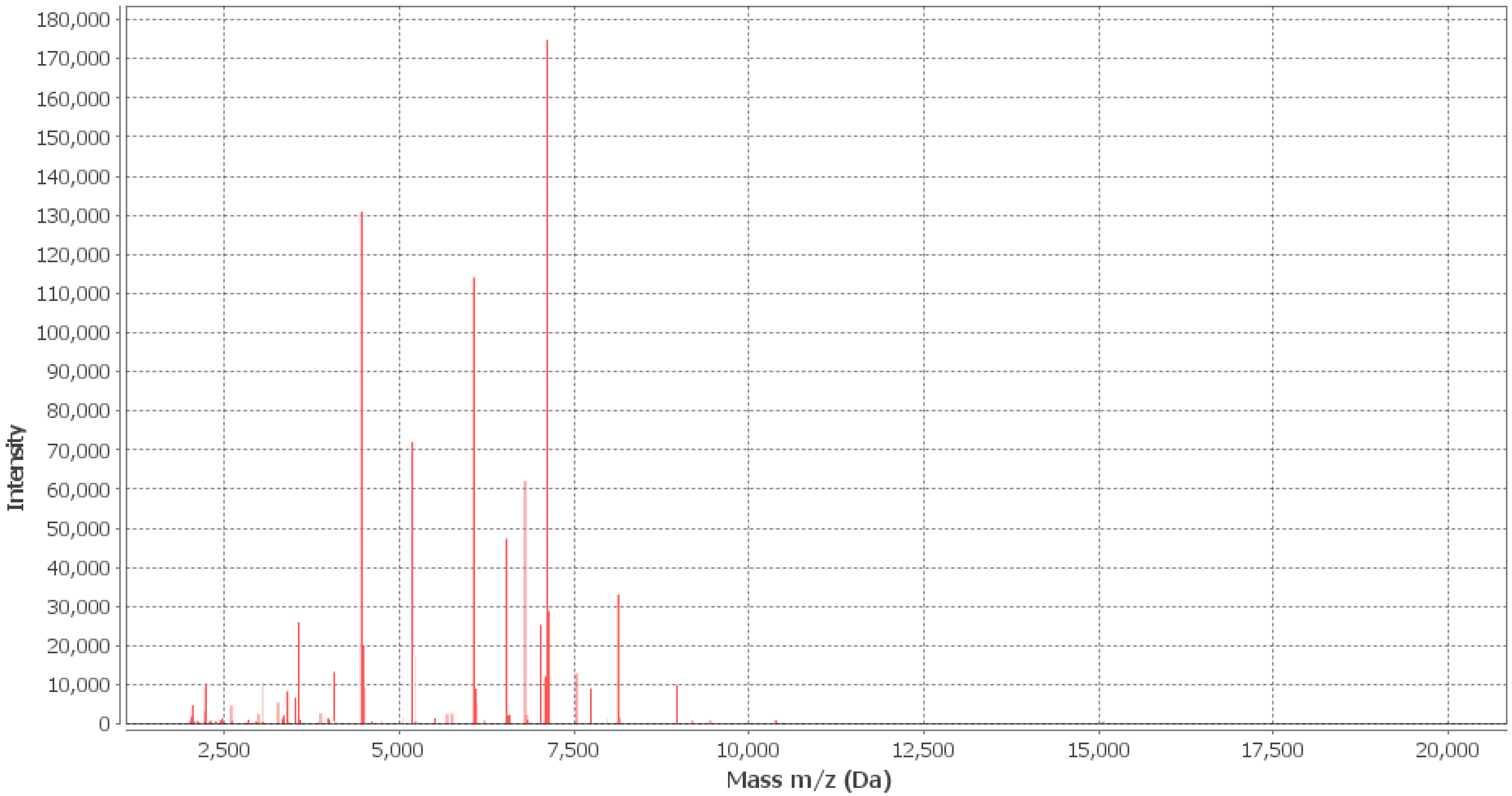

Figure S1. Main spectrum profiles of the *Lactococcus garvieae* isolated during an outbreak in sea bass (*Dicentrarchus labrax*) reared in an inland intensive farm located in the Gulf of Follonica (Tuscany, Italy). Intensity (Y-axis) is shown as a function of the mass-to-charge ratio (m/z, molecular weight for a single positive charge; X-axis).
