# Supplementary material for: The Characterisation of Lactococcus garvieae Isolated in an Outbreak of Septicaemic Disease in Farmed Sea Bass (Dicentrarchus labrax, Linnaues 1758) in Italy
Source: Pathogens. 2024 Jan 4;13(1):49. doi: 10.3390/pathogens13010049 (PMC10820654; doi:10.3390/pathogens13010049)
Supplement: Supplementary file 1 [file pathogens-13-00049-s001.zip › Supplementary_Table_S1_16S_SEQ_cb.pdf]

Table S1 – Salogni et. al – The Characterization of *Lactococcus garvieae* isolated in an Outbreak of Septicaemic Disease in Farmed Sea Bass (*Dicentrarchus labrax*, Linnaues 1758) in Italy

Table S1. Method used for the partial sequencing of 16S ribosomal DNA (rDNA) on strain LI296620A_23 using MicroSEQ 500 16S rDNA Bacterial Identification System (Thermo Fisher Scientific).

Step	Actions and conditions
1	The bacterial suspension was inactivated in PrepMan Ultra Sample Preparation Reagent (Life Technologies) by boiling at 100 °C for 10 min.
2	A 500-bp 16S rDNA fragment was amplified from the 5′ end of the gene in a reaction volume of 30 µl (15 µl of MicroSEQ PCR master mix and 15 µl of bacterial extract) using a MicroSEQ 500 rDNA fast PCR kit (Thermo Fisher Scientific).
3	*The PCR conditions were as follows*: 10 sec of initial denaturation at 95 °C, followed by 30 cycles of 0 sec at 95 °C, 15 sec at 64 °C and 1 min at 72 °C.
4	The amplicon was purified using Exonuclease I and FastAP (Thermo Fisher Scientific).
5	Forward and reverse sequencing reactions were performed on the amplified product using a MicroSEQ 500 rDNA sequencing kit (Thermo Fisher Scientific) following the manufacturer's instructions.
5	The sequencing reactions consisted of 13 µl of MicroSEQ sequencing mix and 7 µl of purified amplified product.
7	The thermal cycling conditions were as follows: 1.30 min at 96 °C, 25 cycles of annealing of 10 sec at 96 °C, 5 sec at 55 °C and 4 min at 60 °C.
8	Sequencing reactions were purified with the BigDye XTerminator Purification Kit (Thermo Fisher Scientific) according to the manufacturer's instructions.
9	The sequence reactions were separated by capillary electrophoresis onto a SeqStudio Genetic Analyzer (Thermo Fisher Scientific) according to the standard automated sequencer protocols.
